# Estimating proportions of explained variance: a comparison of whole genome subsets

**DOI:** 10.1186/1753-6561-8-S1-S102

**Published:** 2014-06-17

**Authors:** Stella Aslibekyan, Howard W Wiener, Guodong Wu, Degui Zhi, Sadeep Shrestha, Gustavo de los Campos, Ana I Vazquez

**Affiliations:** 1 Department of Epidemiology, University of Alabama at Birmingham, 1665 University Blvd, Birmingham, AL 35205, USA; 2 Department of Biostatistics, University of Alabama at Birmingham, 1665 University Blvd, Birmingham, AL 35205, USA

## Abstract

Following the publication of the ENCODE project results, there has been increasing interest in investigating different areas of the chromosome and evaluating the relative contribution of each area to expressed phenotypes. This study aims to evaluate the contribution of variants, classified by minor allele frequency and gene annotation, to the observed interindividual differences. In this study, we fitted Bayesian linear regression models to data from Genetic Analysis Workshop 18 (n = 395) to estimate the variance of standardized and log-transformed systolic blood pressure that can be explained by subsets of genetic markers. Rare and very rare variants explained an overall higher proportion of the variance, as did markers located within a gene rather than flanking regions. The proportion of variance explained by rare and very rare variants decreased when we controlled for the number of markers, suggesting that the number of contributing rare alleles plays an important role in the genetic architecture of chronic disease traits. Our findings lend support to the "common disease, rare variant" hypothesis for systolic blood pressure and highlight allele frequency and functional annotation of a polymorphism as potentially crucial considerations in whole genome study designs.

## Background

The proportion of phenotypic variance explained by genetic factors is influenced by multiple variant attributes. First, an analysis of several complex traits by Yang et al showed that genic regions explain more variation than intergenic because causal variants are more likely to be located in or near the genes, particularly the protein-coding regions [1]. However, insights from the ENCODE project suggest that a number of such regions remain unidentified within the intergenic space and warrant further study [2]. Second, for traits that experience strong selection pressures, rare and very rare mutations (minor allele frequency [MAF] <5%) have been shown to contribute more variance than common mutations [3]. Genome-wide association studies, which rely on linkage disequilibrium between typed and untyped variants, are unlikely to detect effects of rare variants, which can partially explain the majority of variance, yet remain undetected [4]. Although there is clear empirical evidence that variants across the MAF spectrum are important to the genetic architecture of complex traits, there is no consensus on the relative contributions of rare and common polymorphisms to explained variance.

To quantify the relative contribution of subsets of variants (defined by either MAF or functional annotation) to genetic variance of systolic blood pressure (SBP) we fitted Bayesian linear regression models to the Genetic Analysis Workshop 18 (GAW18) data. To control for the effects of the number of variants in each of the subsets, we performed sensitivity analyses with a fixed number of markers in each category to determine the subset with the highest variance contribution per variant.

## Methods

### Phenotypes and covariates

SBP was first log transformed (logSBP) to control skewness and kurtosis. Subsequently, for ease of interpretation, logSBP was standardized to unit variance. To use the available longitudinal data on SBP and covariates and to maximize sample size, a cross-sectional data set was formed by selecting the first available data of 4 visits. Covariates recorded at the time of the visit used for SBP included age, gender, use of tobacco products, and use of medication for blood pressure.

### Estimating variance explained by sets of genetic markers

The whole genome regression models [5] used for analysis were mixed-effects models of the following form: $y_{i}=\mu +\overset{J}{\underset{j=1}{\sum }}{\text{Z}}_{ij}\gamma_{j}+\overset{L}{\underset{l=1}{\sum }}x_{il}\beta_{l}+\varepsilon_{i},$ where $y_{i}$ is the standardized log-transformed blood pressure measured on the *i*^th ^individual (i=1,…,n);*μ *is an intercept, $\overset{J}{\underset{j=1}{\sum }}Z_{ij}\gamma_{j}$ is a regression on nongenetic covariates (sex, age, smoking, and blood pressure medication) whose effects are regarded as fixed; $\overset{L}{\underset{l=1}{\sum }}x_{il}\beta_{l}$ is a regression on marker genotypes $\left\{x_{ij}\right\}$ whose effects $\left\{\beta_{l}\right\}$ are regarded as random; and $\varepsilon_{i}$ are independent, identically distributed normal residuals with mean equal to zero and variance $\sigma_{\varepsilon }^{2}$. Marker genotypes were expressed as deviation from the average genotype, given by 2 times the frequency of the allele coded as 1 at the corresponding marker. Intercept and fixed effects were assigned flat priors; marker effects were assigned independent, identically distributed normal priors with null mean and variance $\sigma_{\beta }^{2}$; and, finally, variance parameters $\left\{\sigma_{\beta }^{2}\sigma_{\varepsilon }^{2}\right\}$ were assigned weakly informative independent scaled-inverted chi-square densities. The algorithms used to implement this model were fully described in prior publications from our group [6]. The software used for the analysis is available upon request. Inferences were based on 25,000 samples obtained after discarding 15,000 as burn-in. Convergence was evaluated by visual inspection of trace plots.

### Variant selection

With the goals of maximizing the biological plausibility of the analysis and reducing the requirements on computational power, we selected a set of 54,309 variants in regions of the genome enriched for blood pressure. The set of variants was identified as follows: We initially selected 84 genes implicated in pathways regulating blood pressure that were included on the SA Biosciences Human Hypertension PCR Array (Qiagen, Venlo, Netherlands). Of the 84 genes reported, only 31 were located on the odd chromosomes and thus available through the GAW18 data release; for our analysis, we selected all variants in or near these 31 genes ( ± 50 kilobases [kb] from upstream or downstream of the start or end sites of transcription, respectively). Table 1 summarizes the distribution of variants near each gene.

**Table 1:** Variants in the hypertension pathway genes located on odd-numbered chromosomes, classified by functional annotation

Gene	Chromosome	Genic variants	Flanking variants
*ADM*	11	30	1423
*ADRA1B*	5	520	4433
*AGT*	1	165	401
*AGTR1*	3	417	12,215
*ATP2C1*	3	1,131	801
*CALCA*	11	54	1521
*CAV1*	7	348	982
*CHRNB1*	17	126	0
*CLIC4*	1	653	1195
*CNGA4*	11	46	93
*DRD3*	3	451	764
*ECE1*	1	1,069	1431
*EDNRB*	13	388	1120
*GCHFR*	15	29	65
*ITPR1*	3	3,567	741
*KNG1*	3	331	744
*MYLK*	3	2,022	183
*NOS3*	7	200	87
*NOSIP*	19	227	145
*NPPB*	1	31	821
*NPR1*	1	123	418
*PDE3B*	11	1,445	19
*PTGS1*	9	299	973
*PTGS2*	1	70	2823
*REN*	1	114	235
*S1PR1*	1	55	2612
*SLC7A1*	13	939	1742
*SPHK1*	17	43	338
*SPHK2*	19	77	0
*UTS2*	1	65	675
*UTS2R*	17	30	244

Subsequently, we excluded variants that did not pass quality control or had more than 2 alleles (*n *= 4470). Variants were further classified on the basis of functional annotation (genic, including intronic, vs. flanking) and allele frequency in the GAW18 data set (very rare if MAF <1%, rare if 1% ≥ MAF ≥5%, common if MAF >5%). To determine functional annotation, we used VCFtools [7] to concatenate all 11 odd-numbered chromosomes' variants and then converted the VCF file to ANNOVAR [8] input format. Using ANNOVAR, we then annotated the variants using Human Genome version 19 (hg19) as a reference, providing gene names (for genic markers) or flanking gene names plus distance to these genes (for markers located in the vicinity of a gene). Variants already reported in dbSNP 131 were also linked with their corresponding rsID numbers. Table 2 shows the number of variants out of our total enriched set (*p *= 49,839) categorized by MAF and functionality. Based on these classifications, we identified 12 sets of markers and fitted whole genome regression models as described above to each set.

**Table 2:** Number of variants categorized by MAF and functionality

Functionality region	All frequencies	Allele frequency		
		
		Common	Rare	Very rare
All regions	49,839 (100%)	11,414 (23%)	6611 (13%)	31,814 (64%)
Genic	16,790 (34%)	2949 (6%)	4763 (10%)	9078 (18%)
Flanking	33,049 (66%)	8465 (17%)	1848 (4%)	22,736 (46%)

Because of variation in the number of variants across the categories in Table 2, it was imperative to assess whether differences in the proportions of variance explained by regression on each of the marker sets were because of the nature of the marker set or the number of markers included in it. To circumvent this problem, we fitted models for each of the marker categories using a fixed number of markers (500) chosen at random from the original set. We fit each of these models 500 times, each time resampling the set of markers used. For each of the models, we quantified the proportion of variance accounted by the model using $R_{m}^{2}=1-{\hat{\sigma }}_{\varepsilon m}^{2}$ where ${\hat{\sigma }}_{\varepsilon }^{2}$ is the estimated residual variance of model *m*. We reported the average of the $R_{m}^{2}$ values over 500 replicate runs of the model. Because the response was standardized to unit variance, $R_{m}^{2}$ is interpretable as an R-squared statistic.

## Results

Tables 3 and 4 give estimates of proportion of variance of logSBP explained by simultaneous regression on nongenetic covariates and sets of markers defined according to functional annotation and MAF. The proportion of phenotypic variance of logSBP explained by regression on all the markers included in the enriched set was estimated at 0.238. In comparison, the model including nongenetic covariates only yielded an estimate of proportion of variance explained of 0.191. Therefore, we conclude that of the total phenotypic variance remaining after accounting for nongenetic effects (0.809 = 1-0.191) roughly 11% (computed as 100 × [0.238-0.191]/[1-0.191]) can be explained by regression on the set of enriched markers. For the models that assessed the role of functional variant annotation, the proportion of variance explained was 0.250 for genic polymorphisms and 0.229 for markers located in flanking regions. The result of the enriched set, which includes variants from both genic and flanking regions, averages over the models that differentiate the regions. For the models that evaluated contributions by allele frequency, the estimates of proportion of variance explained ranged from 0.234 for common variants (which represented 23% of all markers) to 0.259 for very rare variants (64% of all markers) (see Tables 2 and 3). The distribution of variants by MAF was consistent with published observations from other populations [9]. Table 3 shows the estimates of proportion of variance explained when marker sets were defined by functional annotation and MAF. Percentage of variance explained decreased with MAF, regardless of functional annotation. Variants in genic regions generally explained more variance than those in the flanking regions, despite the smaller number of genic markers.

**Table 3:** Proportion of phenotypic variance of log(SBP) explained by simultaneous regression on nongenetic covariates and on marker sets defined based on functional annotation and MAF (analysis without controlling for the number of markers included in each marker set)

Functionality region	All frequencies	Allele frequency		
		
		Common	Rare	Very rare
All regions	0.238	0.234	0.258	0.259
Genic	0.250	0.244	0.254	0.255
Flanking	0.229	0.225	0.245	0.258

**Table 4:** Proportion of phenotypic variance of log(SBP) (averaged over 500 replicates, ± SD) explained by simultaneous regression on nongenetic covariates and on sets of equal size (500 markers), defined according to functional annotation and MAF

Functionality region	All frequencies	Allele frequency		
		
		Common	Rare	Very rare
All regions	0.233 ± 0.054	0.234 ± 0.053	0.252 ± 0.052	0.250 ± 0.063
Genic	0.241 ± 0.053	0.244 ± 0.053	0.253 ± 0.052	0.250 ± 0.063
Flanking	0.227 ± 0.055	0.227 ± 0.054	0.244 ± 0.053	0.246 ± 0.060

Table 4 summarizes the results from the analysis controlling for the number of markers in each category (*p *= 500). Overall, estimates of explained variance slightly decreased compared to those obtained from models including all variants in each category, but the relative contributions of each class of markers remained similar, with rare and very rare variants located in genic regions explaining slightly more outcome variability than common flanking variants.

## Discussion

Using sequence data from a set of 31 biologically relevant genomic regions, we established that the proportion of logSBP variance explained by genetic markers in hypertension-related pathways is roughly 11% of the phenotypic variation that remains after accounting for systematic, nongenetic effects. The proportion of variance explained by regression on markers decreases with the MAF, regardless of whether the variant is located within or near known genes. However, the observed trends across MAF categories were not statistically significant.

To interpret our findings, it is important to distinguish between estimating the proportion of variance explained by the markers, which is the focus of our article, and estimating heritability. Indeed, our estimates of proportion of variance explained by genetic factors (roughly 11% after accounting for differences as a result of nongenetic effects) are likely to be smaller than the true heritability of the trait because, as a result of imperfect linkage disequilibrium between alleles at markers and those at causal loci, some proportion of genetic variance is likely to have remained unaccounted. Our findings are consistent with evidence from several recent studies, which suggest that the genetic architecture of blood pressure is likely to involve a large number of modestly associated, and many yet undiscovered, variants [10-12].

The level of linkage disequilibrium between alleles at markers and those at causal loci depends on linkage disequilibrium decay and on marker density. Consequently, the proportion of variance captured by a maker set is related not only to the nature of the marker set and the genetic architecture of the trait, but also to the number of genetic markers in the set [13]. To account for the effects of the size of different marker sets, we performed an additional analysis, controlling for the number of predictors in each category, enabling direct comparisons between the contributions of very rare, rare, and common variants, as well as between those of flanking and genic markers. We found that the functional annotation of the variants has implications for the proportion of variance explained, especially at higher allele frequencies. However, the relatively small differences in variance explained suggests that flanking regions may harbor yet unidentified genes or regulatory elements that affect polygenic traits. Future studies, particularly of the ENCODE project data, will be informative in testing that hypothesis.

Although our variants were included in the models based on biological relevance, the family structure of the GAW18 data implicates identity by descent as an important contributor to shared genetic variance. To explore other contributions to SBP variance, we conducted sensitivity analyses using only unrelated individuals (data not shown) and found that the basic pattern held true, with rare variants explaining the highest proportion of outcome variance regardless of functional annotation.

As whole genome sequence data become widely available and computational software evolves in its ability to handle a large number of genetic variants, future studies may consider repeating our analyses with a finer classification of markers. Specifically, genic regions could be further subdivided into coding or noncoding regions, or into exonic, intronic, 5′ and 3′ untranslated region, downstream, upstream, and splicing variants. For both genic and flanking regions, another approach would distinguish between variants that encode microRNAs and/or other regulatory molecules, or consider variance explained by differential DNA methylation. Additionally, future studies may evaluate whether the tradeoff between the proportion of outcome variance explained and the multiple testing burden is more favorable for randomly selected (eg, evenly spaced) variants across the genome or for variants located within a priori defined biological pathways. Finally, in our implementation we used shrinkage estimation procedures; however our proposed hypothesis could also be tested using statistical methods that perform variable selection and shrinkage simultaneously. In future, our findings may be extended beyond estimating the proportion of genetic variation to whole genome prediction, fully realizing the potential for clinical and public health applications of deep sequence data.

## Conclusions

We have investigated the tradeoff between proportion of blood pressure variance explained using subsets of the whole genome sequence, and found that rare and very rare variants contribute more outcome variance regardless of their functional annotation.

## Competing interests

The authors declare that they have no competing interests.

## Authors' contributions

SA designed the overall study and drafted the manuscript. HWW, DZ, SS, GC, and AIV developed the analytic strategy and contributed to the interpretation of the data. HWW, GW, DZ, and AIV conducted statistical analyses. GC developed the software used to fit the model and wrote sections of the manuscript. All authors read and approved the final manuscript.
